# Systolic aortic pressure-time area is a useful index describing arterial wave properties in rats with diabetes

**DOI:** 10.1038/srep17293

**Published:** 2015-12-01

**Authors:** Ru-Wen Chang, Chun-Yi Chang, Ming-Shiou Wu, Hsi-Yu Yu, Jian-Ming Luo, Yih-Sharng Chen, Fang-Yue Lin, Liang-Chuan Lai, Chih-Hsien Wang, Kuo-Chu Chang

**Affiliations:** 1 Department of Physiology, College of Medicine, National Taiwan University, Taipei, 100, Taiwan; 2 Department of Emergency Medicine, National Taiwan University Hospital, Chu-Tung Branch, Hsin-Chu, 310, Taiwan; 3 Department of Internal Medicine, National Taiwan University Hospital, Taipei, 100, Taiwan; 4 Department of Surgery, National Taiwan University Hospital, Hsin-Chu Branch, Hsin-Chu, 300, Taiwan; 5 Department of Surgery, National Taiwan University Hospital, Taipei, 100, Taiwan

## Abstract

The accurate measurement of arterial wave properties in terms of arterial wave transit time (*τ*_*w*_) and wave reflection factor (*R*_*f*_) requires simultaneous records of aortic pressure and flow signals. However, in clinical practice, it will be helpful to describe the pulsatile ventricular afterload using less-invasive parameters if possible. We investigated the possibility of systolic aortic pressure-time area (*PTAs*), calculated from the measured aortic pressure alone, acting as systolic workload imposed on the rat diabetic heart. Arterial wave reflections were derived using the impulse response function of the filtered aortic input impedance spectra. The cardiovascular condition in the rats with either type 1 or type 2 diabetes was characterized by (1) an elevation in *PTAs*; and (2) an increase in *R*_*f*_ and decrease in *τ*_*w*_. We found that an inverse linear correlation between *PTAs* and arterial *τ*_*w*_ reached significance (*τ*_*w*_ = 38.5462 − 0.0022 × *PTAs*; *r* = 0.7708, *P* < 0.0001). By contrast, as the *PTAs* increased, the reflection intensity increased: *R*_*f*_ = –0.5439 + 0.0002 × *PTAs*; *r* = 0.8701; *P* <0 .0001. All these findings suggested that as diabetes stiffened aortas, the augmented aortic *PTAs* might act as a useful index describing the diabetes-related deterioration in systolic ventricular workload.

The pulsatile nature of the arterial system is substantially affected by vascular reflections^1^,^2^,^3^. Vascular reflections occur along the entire length of the vasculature anywhere a change occurs in impedance properties. Progressive arterial stiffening causes an accelerated systolic return of pulse wave reflection from the peripheral arterial tree, leading to increased hemodynamic load imposed on the heart^4^,^5^. These changes result in an increased systolic workload and a mismatch in the myocardial supply/oxygen demand ratio, which causes left ventricular (LV) diastolic dysfunction and subsequent systolic dysfunction^4^,^6^.

The accurate measurement of arterial wave properties in terms of arterial wave transit time (*τ*_*w*_) and wave reflection factor (*R*_*f*_) requires simultaneous records of aortic pressure and flow signals. In clinical practice, it will be helpful to describe the arterial wave properties using less-invasive parameters if possible. In 2006, Westerhof *et al.*^7^ provided a noble method to calculate the pressure wave reflection on the basis of the measured aortic pressure alone. Replacing the unknown flow by a triangular wave, they successfully separated the measured pressure wave into its forward and backward components to calculate the reflection magnitude. However, the timing of the reflected wave from the peripheral circulation was not quantified in their study.

Diabetes mellitus (DM) is a complex metabolic disorder^8^,^9^, which is thought to be responsible for impaired hemodynamic load^10^,^11^ and manifests the diabetic cardiomyopathy^12^,^13^. In this study, we investigated the possibility of systolic aortic pressure-time area (*PTAs*) acting as systolic LV workload in rats with diabetes. The aortic *PTAs* was simply calculated from the measured aortic pressure alone. The arterial *τ*_*w*_ was derived to describe the timing of the pulse wave reflection^11^,^14^. The arterial *R*_*f*_ was derived to describe the intensity of the pulse wave reflection^15^. We found that as diabetes stiffened aortas, the aortic *PTAs* was augmented and could reflect the diabetes-related deterioration in arterial wave properties. Because LV relaxation is influenced by hemodynamic load^11^, we also investigated the influence of the aortic *PTAs* on LV myocardial relaxation. Myocardial relaxation was measured indirectly by assessing the time constant of LV isovolumic pressure decay (*τ*_*e*_)^16^. We found that as the aortic *PTAs* increased with diabetes, the LV *τ*_*e*_ became more prolonged and the late pressure relaxation slowed.

## Results

### Exemplification of the recorded pressure and flow signals in one normal rat

**Figure 1:**
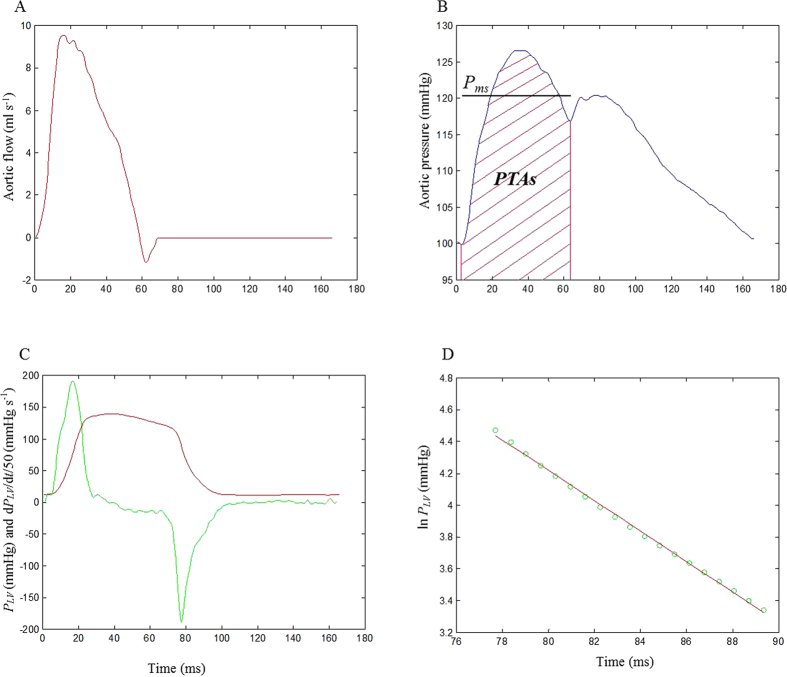
The ascending aortic flow (A), pressure (B), and LV pressure (C) and the calculation of LV *τ*_*e*_ (D) in one normal rat. In (**B**), the red shaded area represents the aortic *PTAs* and the black line is the *P*_*ms*_. The start and end points of systole for *PTAs* calculation were identified as the intersection of 2 tangential lines around the foot of pressure waveform and that around the incisura caused by aortic valve closure, respectively. In (**C**), the red line represents the measured *P*_*LV*_ and the green line is its derivative, i.e., *dP*_*LV*_/*dt*. In (**D**), the time course of LV isovolumic pressure decline is defined by the pressure point of the peak −*dP*_*LV*_/*dt* to 10 mmHg above the end-diastolic pressure. The LV *τ*_*e*_ was calculated as the negative inverse slope of the ln *P*_*LV*_ versus *t* relationship. In this case, the LV *τ*_*e*_ was 8.84 ms with an *r*^*2*^ of 0.9980 and *SEE* of 0.42%. LV, left ventricular; *P*_*LV*_, LV pressure; *P*_*ms*_, mean systolic aortic pressure; *PTAs*, systolic aortic pressure-time area; *r*^*2*^, coefficient of determination; *SEE*, relative standard error of the estimate; *τ*_*e*_, time constant of the LV isovolumic pressure decay.

Figure 1A,B show the measured ascending aortic flow and pressure waveforms, respectively. In Fig. 1B, the red shaded area represents the aortic *PTAs* and the black line is the mean systolic aortic pressure (*P*_*ms*_). Figure 1C,D illustrate the calculation of the LV *τ*_*e*_. The LV *τ*_*e*_ is the inverse negative slope of the ln *P*_*LV*_ versus time (*t*) relation (Fig. 1D); thus, LV *τ*_*e*_ represents the time required for the LV pressure to decrease from a given pressure to 37% thereof. In this case, the LV *τ*_*e*_ was 8.84 ms with an *r*^*2*^ (i.e., the coefficient of determination) of 0.9980 and an *SEE* (i.e., the relative standard error of the estimate) of 0.42%.

**Figure 2:**
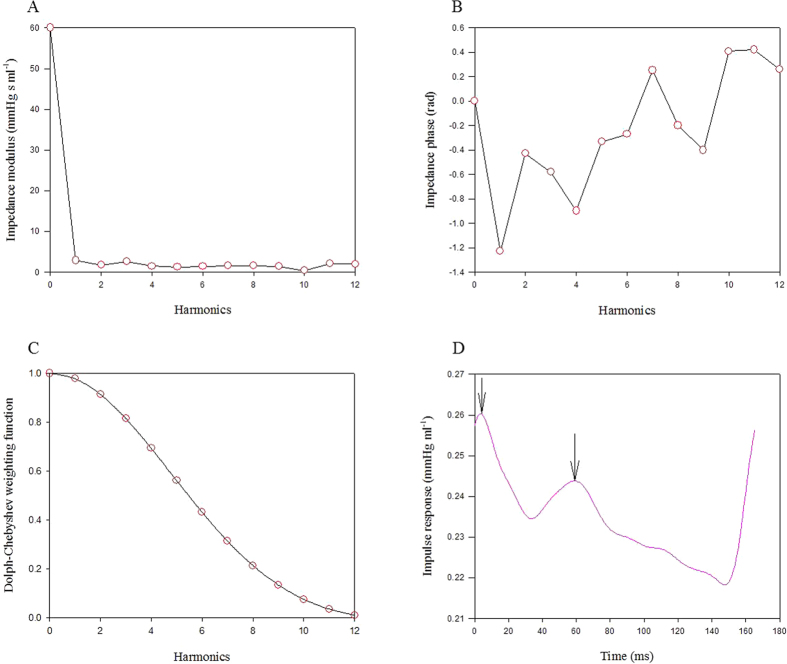
Modulus (A) and phase (B) of the *Z*_*i*_ in the same rat shown in Fig. 1, and a Dolph-Chebychev weighting function with order 24 (C) and the impulse response functin curve (D) derived from the filtered *Z*_*i*_ shown in A and B. In (**C**), this Dolph-Chebyshev filter is used to reduce the effects of truncation of the impedance. In (**D**), the long arrow shows the discrete reflection peak from the body circulation and the short arrow indicates the initial peak as a reference. Half of the time difference between the appearance of the reflected peak and the initial peak approximates the arterial *τ*_*w*_ in the lower body circulation. In this case, the arterial *τ*_*w*_ was 27.9 ms. *Z*_*i*_, aortic input impedance spectra; *τ*_*w*_, wave transit time.

Although the impulse response of the arterial system is the time domain equivalent of its input impedance in the frequency domain, they emphasize different aspects of the system. Figure 2 shows the aortic input impedance (*Z*_*i*_) and its corresponding impulse response of the same normal rat shown in Fig. 1. The impedance modulus fell steeply from a high value at zero frequency (i.e., peripheral resistance) to extremely low values at high frequencies that fluctuated around the aortic characteristic impedance (*Z*_*c*_) (Fig. 2A). The impedance phase indicates the delay between the corresponding pressure and flow components (Fig. 2B). By contrast, Fig. 2D shows the 2 discrete reflection peaks in the impulse response curve, which was calculated through the inverse transformation of *Z*_*i*_ filtered by a Dolph-Chebychev weighting function (Fig. 2C). Half of the time difference between the long and short arrows approximates the arterial *τ*_*w*_ in the lower body circulation. In this case, the arterial *τ*_*w*_ was 27.9 ms.

### Baseline characteristics in diabetes

As expected, after the β-cells of the islets of Langerhans were destroyed by streptozotocin (STZ), the rats with STZ-induced type 1 diabetes had higher blood glucose levels associated with a decrease in body weight (BW) compared with the age-matched controls (NC), as shown in Table 1. Table 1 also shows that partially protected by nicotinamide (NA), the STZ-NA-induced type 2 diabetes yielded moderate and stable hyperglycemia and prevented STZ-induced hypoinsulinemia and BW loss. Both the diabetic groups showed a significant increase in the *PTAs* but not in systolic (*P*_*s*_), diastolic (*P*_*d*_), pulse pressures (*PP*), mean (*P*_*m*_) and *P*_*ms*_ in the aorta. In addition, arterial *R*_*f*_ exhibited a significant increase in both the diabetic groups, with a diabetes-associated reduction in arterial *τ*_*w*_.

Regarding the LV pressure profile, the rats with type 1 (but not type 2) diabetes had higher LV end-diastolic pressure (*P*_*ed*_) and lower −*dP*_*LV*_/*dt*, as shown in Table 2. By contrast, the peak LV pressure did not change significantly as the rats developed hyperglycemia in both the diabetic groups. However, a diabetes-associated increase in LV *τ*_*e*_ was noted. The linearity of the ln *P*_*LV*_ versus *t* relation was reported as *r*^*2*^, and was higher than 0.9950 with an *SEE* lower than 1.0% in each group.

### Association of the aortic *PTAs* with arterial *R*_*f*_ and *τ*_*w*_ and LV *τ*_*e*_

**Figure 3:**
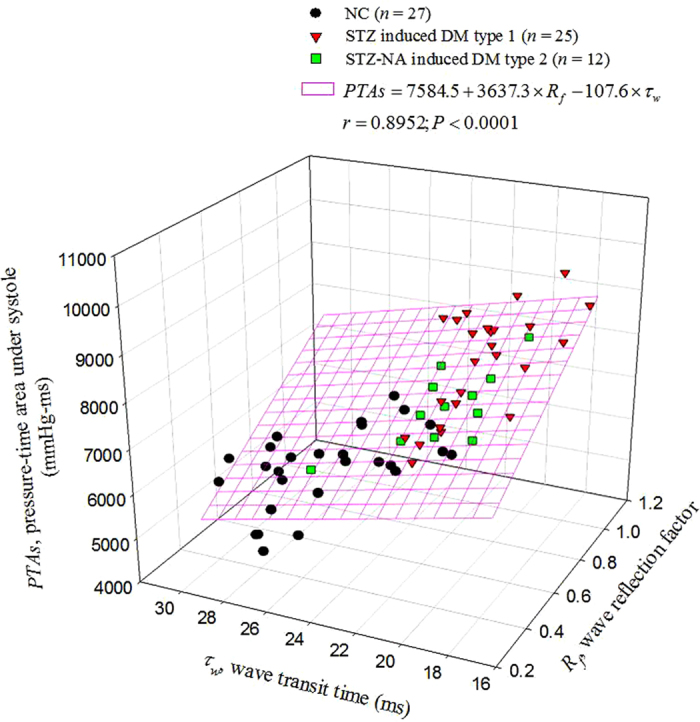
Implication of arterial wave properties in *PTAs*. As shown by multiple linear regression analysis, the correlation between the aortic *PTAs* and the arterial *τ*_*w*_ and *R*_*f*_ reached significance, suggesting that the arterial wave properties impaired by diabetes could be reflected in the aortic *PTAs*. *PTAs*, systolic aortic pressure-time area; *R*_*f*_, wave reflection factor; *τ*_*w*_, wave transit time.

**Figure 4:**
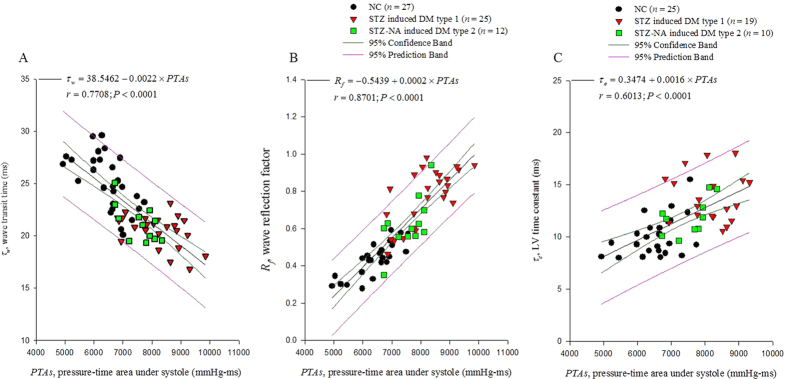
Potential role of aortic *PTAs* in reflecting arterial wave properties and predicting LV isovolumic pressure relaxation. The arterial *τ*_*w*_ was significantly inversely related to the *PTAs* (**A**). By contrast, as the *PTAs* increased, the reflection intensity (arterial *R*_*f*_) increased (**B**). A positive linear correlation existed between the LV *τ*_*e*_ and aortic *PTAs* (**C**), indicating that in diabetes, the *PTAs* increases and the prolonged LV *τ*_*e*_ slows the late pressure relaxation. LV, left ventricular; *PTAs*, systolic aortic pressure-time area; *R*_*f*_, wave reflection factor; *τ*_*e*_, time constant of the LV isovolumic pressure decay; *τ*_*w*_, wave transit time.

By taking *PTAs* as the dependent variable and arterial *R*_*f*_ and *τ*_*w*_ as the two independent variables, multiple linear regression shown in Fig. 3 exhibited a favorable correlation among the three parameters (*PTAs* = 7584.5 + 3637.3 × *R*_*f*_  – 107.6 × *τ*_*w*_; *r* = 0.8952, *P* < .0001). Figure 4 shows the ability of *PTAs* to predict arterial wave properties and LV isovolumic pressure relaxation in diabetes. The inverse linear correlation between *PTAs* and arterial *τ*_*w*_ reached significance (*τ*_*w*_ = 38.5462 – 0.0022 × *PTAs*; *r* = 0.7708, *P* < .0001) (Fig. 4A). By contrast, *PTAs* had positive linear correlation with the arterial *R*_*f*_ : *R*_*f*_ = –0.5439 + 0.0002 × *PTAs*; *r* = 0.8701; *P* < .0001 (Fig. 4B). Moreover, the significant linear correlation between LV *τ*_*e*_ and *PTAs* was noted (*τ*_*e*_ = 0.3474 + 0.0016 × *PTAs*; *r* = 0.6013, *P* < .0001) (Fig. 4C).

## Discussion

In 1975, Milnor^17^ has emphasized the role of proximal aortic impedance in the ventricular afterload. However, the *Z*_*i*_ is difficult to obtain in clinical setting, because it is calculated from the simultaneously recorded aortic pressure and flow signals by using Fourier analysis^2^,^3^. In this study, we demonstrated the implication of arterial wave properties in aortic *PTAs* as a hemodynamic load imposed on the heart, which can influence the LV isovolumic pressure relaxation in rats with either type 1 or type 2 diabetes.

Arterial stiffening determines the arterial pressure shape and amplitude, influencing systolic, diastolic, and pulse pressures in the aorta^4^,^18^. According to the Moens and Korteweg formula^19^, pulse wave velocity (*c*_*0*_) may be approximately related to the elastic incremental modulus of the arterial wall (*E*_*i*_): 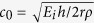 where *ρ* is the blood density and *h*/*2r* is the ratio of wall thickness to the lumen diameter. This formula indicates that as the arterial stiffness increases (i.e. increased *E*_*i*_), *c*_*0*_ increases and thereby shortens travelling time of the forward and reflected pressure waves. With increased *c*_*0*_, the reflected pressure wave returns earlier, which impacts on the central arteries during systole rather than diastole, amplifies aortic and ventricular pressures during systole, and reduces aortic pressure during diastole. Such alterations create an increased systolic workload and a mismatch in the myocardial supply/oxygen demand ratio^6^, which may cause cardiac failure and cardiovascular death in patients with diabetes, hypertension and end-stage renal disease^20^,^21^,^22^.

Arterial stiffness can be measured with several methods depending on the clinical use or experimental situation^23^,^24^. In this study, arterial *τ*_*w*_, which is inversely related to *c*_*0*_, was derived to represent the distensibility of aortas; the stiffer the aortic wall, the shorter the arterial *τ*_*w*_, and vice versa^2^,^3^. Rats with either DM type 1 or type 2 showed an increase in aortic stiffness compared with the NC, as evidenced by a reduction in *τ*_*w*_ (Table 1). A reduction in *τ*_*w*_ suggested that diabetes caused an early return of pulse wave reflection from the peripheral circulation. Diabetes also contributed to a significant increase in arterial *R*_*f*_, augmenting the reflection intensity. These findings were congruent with the previous findings that early return of the enhanced pulse wave reflection was frequently observed in patients with diabetes^25^,^26^.

As mentioned, the pulsatile nature of arterial pressure is substantially affected by arterial distensibility and the timing and intensity of the wave reflection. Although both DM type 1 and type 2 stiffened aortas and shortened arterial *τ*_*w*_, both the diabetic groups exhibited no significant changes in *P*_*s*_, *P*_*d*_, *PP*, *P*_*m*_ and *P*_*ms*_ (Table 1). By contrast, the aortic *PTAs* augmented by diabetes was associated with the impaired arterial wave properties. Using multiple linear regression analysis, we found that the aortic *PTAs* was affected by the timing and magnitude of pulse wave reflection, for arterial *τ*_*w*_ and arterial *R*_*f*_ (Fig. 3). As arterial *τ*_*w*_ shortened and arterial *R*_*f*_ was augmented with diabetes, the aortic *PTAs* became larger. In other words, arterial *τ*_*w*_ was significantly inversely affected by *PTAs* (Fig. 4A). By contrast, as *PTAs* increased, the reflection intensity (arterial *R*_*f*_) increased (Fig. 4B). These findings suggest that the systolic loading condition for the left ventricle coupled to the arterial system could be implicated in the aortic *PTAs*.

Research has established that LV relaxation is influenced by the hemodynamic loads imposed on the heart^27^,^28^,^29^,^30^,^31^. As mentioned, the diabetes-related cardiovascular dynamic changes in rats were characterized by impaired arterial wave properties and LV *τ*_*e*_ (Table 1). Because the aortic *PTAs* had the ability to reflect alterations in the pulsatile ventricular afterload, we investigated the association of LV *τ*_*e*_ with aortic *PTAs* in rats with diabetes. We found a positive linear correlation between LV *τ*_*e*_ and aortic *PTAs* (Fig. 4C), indicating that in diabetes, *PTAs* increases and the prolonged LV *τ*_*e*_ slows the late pressure relaxation. These results were consistent with the findings of other studies^26^,^32^; in systolic maximal loading conditions caused by an impaired aortic elastic function, abnormalities in the LV diastolic function occurred in patients with diabetes, contributing to the development of diabetic cardiomyopathy.

This study had several limitations. Because the *Z*_*i*_ cannot be measured in conscious animals, evaluating the effects of pentobarbital anesthesia on rats is impossible. The results reported here pertain only to the measurements made in anesthetized rats in the open-chest condition. This condition might have induced changes in the aortic pressure profiles and introduced reflex effects not found in the closed-chest condition. The degree to which anesthesia and thoracotomy influence the pulsatile hemodynamics in rats is uncertain. However, studies with other animal models suggest that the effects are small relative to the biological and experimental variability between animals^33^.

The principle finding of this study is that the arterial wave properties could be implicated in the aortic *PTAs* in diabetes; as the aortic *PTAs* increased, the arterial *τ*_*w*_ shortened and the reflection intensity (arterial *R*_*f*_) increased. We also found a positive linear correlation between LV *τ*_*e*_ and aortic *PTAs*, indicating that in diabetes, *PTAs* increases and the prolonged LV *τ*_*e*_ slows the late pressure relaxation. All these findings suggested that the aortic *PTAs*, simply calculated from the measured pressure alone, might act as systolic LV workload and influence the LV isovolumic pressure decay in rats with diabetes. In this study, we provided a foundation for considering the clinical application of aortic *PTAs* in evaluation of the pulsatile ventricular afterload and LV myocardial relaxation.

## Methods

### Animals and catheterization

Two-month-old male Wistar rats were randomly divided into 3 groups, as follows: (1) NC (*n* = 27), (2) type 1 DM (*n* = 25), and (3) type 2 DM (*n* = 12). Type 1 DM was induced by using a single tail-vein injection with 55 mg kg^−1^ of STZ (Sigma, St. Louis, MO, USA) in a 0.1 *M* citrate buffer (pH 4.5) (Sigma, St. Louis, MO, USA)^13^. Type 2 DM was induced by administering intraperitoneally 180 mg kg^−1^ of NA (Sigma, St. Louis, MO, USA) 30 min before an intravenous injection of 50 mg kg^−1^ of STZ dissolved in 0.1 *M* citrate buffer (pH 4.5)^34^,^35^. The blood glucose level was determined using a SURESTEP Test Strip (Lifescan Inc., Milpitas, CA, USA) to confirm the development of hyperglycemia. Studies on the changes in cardiovascular mechanics were performed 8 weeks after the induction of diabetes. All rats were allowed free access to Purina Chow and water with a 12-hour light/dark cycle. The experiments were conducted according to the *Guide for the Care and Use of Laboratory Animals*, and our study protocol was approved by the Animal Care and Use Committee of the National Taiwan University.

The general surgical procedures and method used to measure the cardiovascular variables in the anesthetized rats were as described previously^11^. In brief, the animals were anesthetized using intraperitoneal sodium pentobarbital (50 mg kg^−1^), placed on a heating pad, intubated, and ventilated with a rodent respirator (Model 131, New England Medical Instruments, Medway, MA, USA). The chest was opened through the second intercostal space on the right side. An electromagnetic flow probe (Model 100 series, internal circumference 8 mm, Carolina Medical Electronics, King, NC, USA) was positioned around the ascending aorta to measure the pulsatile aortic flow. A high-fidelity pressure catheter (Model SPC 320, size 2F, Millar Instruments, Houston, TX, USA) was used to measure the pulsatile aortic pressure through the isolated carotid artery on the right side, and then advanced into the left ventricle to record the LV pressure wave. The electrocardiogram (ECG) of lead II was recorded using a Gould ECG/Biotach amplifier (Cleveland, OH, USA). The selective aortic pressure and flow signals from 5−10 beats were averaged in the time domain by using the peak R-wave of the ECG as a fiducial point. The timing asynchronicity between the pressure and flow signals caused by the spatial distance between the flow probe and the proximal aortic pressure transducer was corrected using a time-domain approach, in which the foot of the pressure waveform was realigned with that of the flow^36^. The resulting aortic pressure and flow signals were subjected to further vascular impedance analysis. The selective LV pressure signals from 5−10 beats were averaged in the time domain to calculate the LV *τ*_*e*_^16^.

### Aortic input impedance spectra and impulse response function curve

The *Z*_*i*_ was obtained from the ratio of ascending aortic pressure harmonics to the corresponding flow harmonics by using a standard Fourier series expansion technique^2^,^3^,^11^, shown in Appendix 1. The *Z*_*c*_ was computed by averaging the high-frequency moduli of the impedance data points (4th−10th harmonics). The arterial *τ*_*w*_ was computed using the impulse response function of the filtered *Z*_*i*_^37^,^38^. This calculation was performed through the inverse transformation of *Z*_*i*_ after multiplying the first 12 harmonics by a Dolph-Chebychev weighting function with order 24^14^, shown in Appendix 2. The arterial *R*_*f*_ was calculated as the amplitude ratio of backward-to-forward peak pressure waves, by using the method proposed by Westerhof *et al.*^15^, shown in Appendix 3. Therefore, both the arterial *τ*_*w*_ and *R*_*f*_ characterized the wave reflection as it occurred in the rat vasculature.

### Time constant of the LV Isovolumic pressure decay

The LV end-diastolic point was identified as the peak of the ECG R-wave. The time course of LV isovolumic pressure decay was defined by the pressure point of the peak 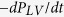 to 10 mmHg above the *P*_*ed*_. The LV *τ*_*e*_ was calculated as follows^16^:


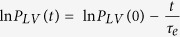


ln* P*_*LV*_(0) is the pressure intercept at zero time point, and *τ*_*e*_ is the time constant of the LV isovolumic exponential pressure decline, which is the inverse negative slope of the ln* P*_*LV*_ versus *t* relation. Because the LV isovolumic pressure decay was assumed to be monoexponential, we examined the linearity of the ln* P*_*LV*_ versus *t* relation and calculated LV *τ*_*e*_ only when the relation between ln *P*_*LV*_ and *t* yielded a high linear correlation coefficient. The linearity of the ln* P*_*LV*_ versus *t* relation was reflected in the *r*^*2*^ and the *SEE* calculated from the linear regression between ln* P*_*LV*_ and *t.*

### Statistics

Results are expressed as means ± standard error (s.e.). One-way analysis of variance (ANOVA) was performed to determine the statistical significance of the results for multiple comparisons of the effect of diabetes on arterial wave properties and LV myocardial relaxation. Statistical significance was assumed at the level of *P* < 0.05. Where the ANOVA results indicated that a hemodynamic variable differed significantly in different groups, the Tukey’s honestly significant difference (HSD) method was used to determine the groups of rats that obtained divergent mean values for that variable.

### Appendix 1

#### Mathematic consideration for the aortic input impedance analysis

In the study of *Z*_*i*_ using Fourier series analysis, a discrete-time linear shift invariant system must be applied to the systemic arterial circulation. The physical properties of the arterial system is then completely characterized by impulse response *z*_*i*_[*n*], if taking measured aortic flow *q*[*n*] as the input and measured aortic pressure *p*[*n*] as the output, *n* = 0, 1, 2, 3, … , *N* – 1. The fundamental expression of this input-output relationship is known as the convolution sum:


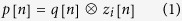


where 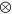 is the convolution operator. According to the convolution theorem^39^, with frequency response *Z*_*i*_[*k*], the relationship between the aortic pressure and aortic flow can be written as follows:


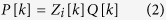


where *P*[*k*], *Q*[*k*], and *Z*_*i*_[*k*] are the Fourier transforms of *p*[*n*], *q*[*n*] and *z*_*i*_[*n*], respectively. The discrete Fourier transforms of the measured signals *p*[*n*] and *q*[*n*] are defined by the equations


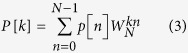



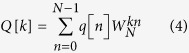


where *k* = 0, 1, 2, 3, … , *N* – 1, 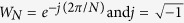. For *k*^*th*^ sinusoidal signal, the *Z*_*i*_[*k*] is the ratio of ascending aortic pressure harmonic to the corresponding flow harmonic:


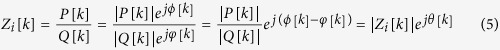


where 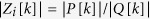 is the modulus and 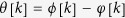 is the phase of the impedance. In this study, the level of the flow noise was determined by Fourier analysis of the middle third of the diastolic flow signal^40^. Any flow harmonic with a modulus < 1.5 times the noise level was not used for impedance calculation.

### Appendix 2

#### Dolph-Chebychev weighting function

The impulse response of the arterial system is the response resulting from a flow that is a unit impulse function (i.e., infinitely short in duration and infinitely high with unit area). In clinical practice and experimental situation, using a flow impulse as an excitation to the vasculature is difficult to carry out. Therefore, the impulse response of the arterial system is calculated via inverse Fourier transform of the input impedance. However, this calculation contains spurious oscillations introduced by the truncation of the input impedance data. In 1978, Laxminarayan *et al.*^14^ introduced a Dolph-Chebyshev filter to reduce the effects of truncation of the impedance. The Chebyshev polynomial of the first kind and order *p* is defined by a recurrent relationship:


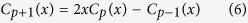


with *C*_*0*_ = 1 and *C*_*1*_ = *x*. They introduced a new variable modified with respect to Dolph’s variable, which is directly related to the harmonic number:


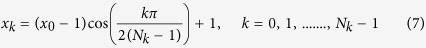


where *k* is the harmonic number and *N*_*k*_ are the number of harmonics available in the interval (−*x*_*0*_, −1). The order of the polynomial *p* is chosen to be 2*N*_*k*_. The ripple factor is defined as the ratio of the function at *x* = −*x*_0_ and *x* = −1. Normalization is then performed by dividing all polynomial values by the ripple factor. This modified Dolph-Chebyshev weighting function contains most of the energy in the main lobe and has relatively small side lobe in the calculation of impulse response function.

### Appendix 3

#### Arterial wave reflection factor

In the time domain, the measured aortic pressure *p*(*t*) and flow *q*(*t*) waves can be dissected into their forward (or incident) and backward (or reflected) components:


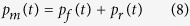



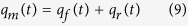


The subscripts *m*, *f*, and *r* indicate measured, forward and reflected, respectively. If one considers an idealized system in which there are no wave reflections, one can define the *Z*_*c*_ as follows:


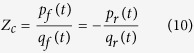


Assuming that *Z*_*c*_ is a real number, the forward and backward components of the aortic pressure and flow signals can be calculated, the formula of which are


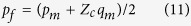



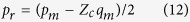



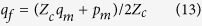



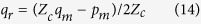


From the definition of *R*_*f*_, we have:


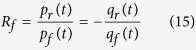


In this study, the time domain reflection factor was calculated as the amplitude ratio of backward-to-forward peak pressure waves, proposed by Westerhof *et al.*^15^.

## Additional Information

**How to cite this article**: Chang, R.-W. *et al.* Systolic aortic pressure-time area is a useful index describing arterial wave properties in rats with diabetes. *Sci. Rep.* **5**, 17293; doi: 10.1038/srep17293 (2015).

## Figures and Tables

**Table 1:** Effects of diabetes on the blood glucose level, body weight, aortic pressure profile, and arterial wave properties of male Wistar rats.

Group	NC (*n* = 27)	DM type 1 (*n* = 25)	DM type 2 (*n* = 12)
BS (mg dl^−1^)	101.0 ± 1.9	459.4 ± 13.7*	159.8 ± 23.5†
BW (g)	452.0 ± 5.9	301.9 ± 5.5*	420.8 ± 12.2
*HR* (beats/min)	408.1 ± 6.1	354.9 ± 4.8*	368.9 ± 10.2†
*P*_*s*_(mmHg)	117.4 ± 2.3	113.6 ± 2.0	124.9 ± 4.2
*P*_*d*_ (mmHg)	93.8 ± 2.4	88.8 ± 2.4	101.6 ± 3.5
*PP* (mmHg)	23.6 ± 0.5	24.8 ± 1.0	23.3 ± 1.1
*P*_*m*_ (mmHg)	106.4 ± 2.4	102.3 ± 2.1	114.8 ± 3.9
*P*_*ms*_ (mmHg)	111.3 ± 2.4	107.4 ± 2.0	119.6 ± 4.1
*PTAs* (mmHg-ms)	6469.2 ± 139.8	8188.9 ± 170.9*	7586.6 ± 164.1†
*CO* (ml s^−1^)	2.00 ± 0.08	2.10 ± 0.09	1.69 ± 0.09†
*τ*_*w*_(ms)	25.2 ± 0.5	20.5 ± 0.3*	21.2 ± 0.5†
*R*_*f*_	0.44 ± 0.02	0.77 ± 0.03*	0.62 ± 0.04†

All values are expressed as means ± s.e. BS, blood sugar; BW, body weight; *HR*, basal heart rate; *P*_*s*_, systolic aortic pressure; *P*_*d*_, diastolic aortic pressure; *P*_*m*_, mean aortic pressure; *P*_*ms*_, mean systolic aortic pressure; *PP*, pulse pressure; *PTAs*, systolic aortic pressure-time area; *CO*, cardiac output; *τ*_*w*_, wave transit time; *R*_*f*_, wave reflection factor; NC, normal controls; DM type 1, STZ-induced diabetic rats; DM type 2, STZ-NA-induced diabetic rats.

^*^P < 0.05 when the DM type 1 was compared with the NC.

^†^*P* < 0.05 when the DM type 2 was compared with the NC.

**Table 2:** Effects of diabetes on the LV pressure profile and LV isovolumic pressure relaxation of male Wistar rats.

Group	NC (*n* = 25)	DM type 1 (*n* = 19)	DM type 2 (*n* = 10)
*P*_*ed*_(mmHg)	4.10 ± 0.68	7.65 ± 1.3*	5.50 ± 1.35
*P*_*LVP*_ (mmHg)	127.9 ± 3.0	121.2 ± 2.2	121.8 ± 4.2
−*dP*_*LV*_/*dt* (mmHg s^−1^)	−6960.0 ± 300.2	−5627.0 ± 253.1*	−5972.9 ± 325.4
*τ*_*e*_ (ms)	10.0 ± 0.4	13.8 ± 0.5*	12.0 ± 0.5†
*r*^*2*^	0.9975 ± 0.0005	0.9975 ± 0.0010	0.9985 ± 0.0004
*SEE* (%)	0.540 ± 0.062	0.437 ± 0.086	0.406 ± 0.056

All values are expressed as means ± s.e. LV, left ventricular; *P*_*ed*_, LV end-diastolic pressure; *P*_*LV*_, LV pressure; *P*_*LVP*_, peak LV pressure; *τ*_*e*_, time constant of the LV isovolumic pressure decay; *r*^*2*^, coefficient of determination; *SEE*, relative standard error of the estimate; NC, normal controls; DM type 1, STZ-induced diabetic rats; DM type 2, STZ-NA-induced diabetic rats.

^*^P < 0.05 when the DM type 1 was compared with the NC.

^†^*P* <0.05 when the DM type 2 was compared with the NC.
